# Dyslipidaemia and inflammatory markers as the risk predictors for cardiovascular disease in newly diagnosed premenopausal hypothyroid women

**DOI:** 10.5937/jomb0-37007

**Published:** 2023-01-20

**Authors:** Vaideki Balamurugan, Ravindra Maradi, Vivek Joshi, Belle Vijetha Shenoy, Manjunatha B.K. Goud

**Affiliations:** 1 Manipal Academy of Higher Education, Kasturba Medical College, Manipal, India; 2 Drexel University College of Medicine, Wyomissing, PA, USA; 3 RAK Medical and Health Sciences University, RAKCOMS, Ras Al Khaimah, UAE

**Keywords:** hypothyroidism, hs-CRP, dyslipidemia, atherogenic indices, cardiovascular disease, Framingham Risk Score, homocysteine, hipotireoza, hs-CRP, dislipidemija, aterogeni indeksi, kardiovaskularne bolesti, Framinghamski skor rizika, homocistein

## Abstract

**Background:**

Hypothyroidism can predispose systolic and diastolic cardiac dysfunction, increased peripheral vascular resistance, endothelial dysfunction, altered coagulopathy, and dyslipidemia resulting in atherosclerosis. Thyroid hormones can influence homocysteine metabolism by regulating the methylenetetrahydrofolate reductase (M THR). So, this study aimed to compare the markers homocysteine, high sensitive C-reactive protein (hs-CRP), and Atherogenic Indices (AI) between newly diagnosed hypothyroid and euthyroid premenopausal women.

**Methods:**

80 Female patients between 20 and 45 years were enrolled in this study and were equally divided into cases and controls group. Laboratory tests included: i) Serum T3, T4, TSH was measured using electrochemiluminescence, ii) lipid profile, homocysteine, and hs-CRP were measured for all the participants. Atherogenic indices: Castelli risk indices I&II, Atherogenic coefficient (AEC), and Atherogenic Index of Plasma (AIP) were calculated using formulas. A comparison between the study groups was made by using the Independent t-test and Mann-Whitney U test. p-value < 0.05 was considered significant.

**Results:**

The hypothyroid group had significantly higher levels of homocysteine (p= 0.014), and hs-CRP (hs-CRP> 3.0 mg/L, 70% of participants have intermediate to high risk for a cardiovascular event) and elevated BMI compared to participants in the euthyroid group. Atherogenic indices (p< 0.001) was significantly increased in the hypothyroid participants' group. TC, TG , and LDL were significantly elevated in the hypothyroid group but did not show any association with systolic and diastolic blood pressure.

**Conclusions:**

Premenopausal women with hypothyroidism have a greater predisposition for cardiovascular disease compared to euthyroid

## Abstract (sr)

**Uvod:**

Hipotireoza može da predisponira sistolnu i dijastolnu srčanu disfunkciju, povećan periferni vaskularni otpor, endotelnu disfunkciju, izmenjenu koagulopatiju i dislipidemiju koja dovodi do ateroskleroze. Hormoni štitne žlezde mogu da utiču na metabolizam homocisteina regulacijom metilentetrahidrofolat reduktaze (MTHR). Dakle, ova studija je imala za cilj da uporedi markere kao što su homocistein, visoko osetljivi C-reaktivni protein (hs-CRP) i Aterogene indekse (AI) kod žena sa novodijagnostikovanom hipotireozom i eutireozom u premenopauzi.

**Metode:**

U ovu studiju je bilo uključeno 80 pacijentkinja starosti između 20 i 45 godina koje su podjednako podeljene na ispitanice i kontrolnu grupu. Laboratorijski testovi su uključivali: i) serumski T3, T4, TSH koji su mereni elektrohemiluminiscencijom, ii) lipidni profil, homocistein i hs-CRP koji su mereni za sve učesnike. Aterogeni indeksi: Kastelijevi indeksi rizika I & II, Aterogeni koeficijent (AEC) i Aterogeni indeks plazme (AIP) koji su izračunati korišćenjem formula. Poređenje između ispitivanih grupa je urađeno korišćenjem nezavisnog t-testa i Mann-Whitney U testa sa značajnom p-vrednoš u od < 0.05.

**Rezultati:**

Hipotiroidna grupa je imala značajno više nivoe homocisteina (p = 0,014) i hs-CRP (hs-CRP > 3,0 nmol/L, 70% učesnica je imalo srednji do visok rizik od diovaskularnih događaja) i povišen BMI u poređenju sa učesnicima u eutireoidnoj grupi. Aterogeni indeksi (p < 0,001) su bili značajno povećani u grupi učesnica sa hipotireozom. TC, TG i LDL su bili značajno povišeni u hipotiroidnoj grupi, ali nisu pokazali nikakvu povezanost sa sistolnim i dijastolnim krvnim pritiskom.

**Zaključak:**

Žene u premenopauzi sa hipotireozom imaju veću predispoziciju za kardiovaskularne bolesti u poređenju sa pacijentkinjama sa eutireozom.

## Introduction

The World Health Organization (WHO) considers cardiovascular diseases (CVD) the most common cause of death worldwide, accounting for around 17.9 million deaths globally, more than two-thirds of which are in developing countries. CVD is a constellation of disorders involving coronary heart diseases, cerebrovascular diseases, rheumatic heart diseases, and others. Lack of physical activity, smoking, alcohol ingestion, and an unhealthy diet are important risk factors in developing CVD and are often associated with other pathological conditions like diabetes mellitus, thyroid disorders, renal dysfunction, hypertension, and dyslipidemia [1] [2]. Previous studies have established a positive association between thyroid abnormality, homocysteine concentration in blood, systemic inflammatory processes, and cardiovascular diseases [3] [4].

Thyroid hormones have a vital role in maintaining the homeostasis of the cells, and its abnormalities can be classified into hypothyroidism and hyperthyroidism. Hypothyroidism can be further divided into overt hypothyroidism (OvertHO) and subclinical hypothyroidism (SCH) based on the levels of serum thyroid-stimulating hormone and serum thyroxine [5]. Hypothyroidism is characterized by low basal metabolic rate, heat generation, and oxygen expenditure. The primary mechanism by which the thyroid hormone disorder can predispose to systolic and diastolic heart failure is by altering cardiac myocyte contractility and surface receptor-mediated remodelling of the cardiomyocyte [6] [7]. There are conflicting views about hypothyroidism being an independent risk factor for developing cardiovascular pathology or mortality or as part of a group of risk factors [8].

Dyslipidaemia has been strongly associated with an increased risk of developing CVD. The major characteristics of dyslipidaemia observed in cases with cardiovascular events, which are all part of atherogenic indices, were hypercholesterolemia, hypertriglyceridemia, elevated LDL, decreased HDL, and increased lipoprotein(a) [9]. It is recommended that the multiple biochemical parameters be utilized while predicting the risk for coronary vascular diseases, but not individually; the various mathematically calculated indices used by the clinicians for predicting the risk for CVDs are the Atherogenic index of plasma (AIP), Castelli Risk Index (CRI), and Atherogenic Coefficient (AC) [10] [11].

Prolonged dyslipidaemia can stimulate pro-inflammatory mechanisms in humans and can lead to the release of cytokines and other inflammatory markers. High-sensitive C-reactive protein (hs-CRP) is one of the most critical and reliable biochemical markers for the presence of systemic inflammation. It is overly sensitive in predicting the impending vascular events or markers following any cardiovascular events or systemic infections [12].

Homocysteine is a common non-proteinogenic amino acid found in blood and is synthesized from methionine during biochemical metabolisms. This homocysteine can be recycled back into methionine or converted into cysteine by vitamin B6, B12, and folic acid. Any derangement in thyroid hormone status can alter the homocysteine metabolism by reducing the expression of the enzyme methylenetetrahydrofolate reductase (MTHFR) and methionine synthase in the liver. This results in hyperhomocysteinemia. Elevated levels of homocysteine in the blood can lead to premature hardening and irritation of blood vessels, which could eventually predispose to cardiovascular events in young adults [12] [13].

The National Health Nutrition Examination Survey III (NAHES III) in the United States revealed a 4.6% prevalence of hypothyroidism and a 4.3% prevalence of subclinical hypothyroidism, with a higher prevalence in women and the elderly [5]. Other studies in Europe have determined that the prevalence of undiagnosed hypothyroidism was 4.94%, with a considerable female preponderance of 8.12%. A similar trend was also observed in the Indian population, hypothyroidism was prevalent in 10.95% of Indian adults, with a higher prevalence rate in females (15.86 %) than males and a higher prevalence rate in older adults (46–54 years) (13.11 %) [6] [7].

As described in the previous studies, hypothyroidism is common in young adult females in the premenopausal age group. This can have a prolonged and lasting effect on the cardiovascular system. Premenopausal age is the time between the woman’s first menstruation and the perimenopausal phase. As part of routine investigation, hs-CRP and homocysteine have never been measured in premenopausal females with clinical hypothyroidism. This research aimed to investigate the thyroid hormone dysfunctionality in the form of dyslipidaemia, hyperhomocysteinemia, and altered hs-CRP in newly diagnosed hypothyroid women in premenopausal age to evaluate whether these biomarkers could be used as screening markers in newly diagnosed hypothyroid women to predict the CVD risk.

## Materials and methods

### Study design and participants

This is a single-center cross-sectional, case-control study on the patients enrolled at Kasturba Medical College & Kasturba Hospital, Manipal University, Manipal, India. The study included 80 subjects, divided into two separate categories; 40 participants were grouped as cases and 40 in the control group. The participants were enrolled in the study following permission from the Institutional Ethics Committee (I.E.C 644/2016) and after obtaining written consent. A female patient recently diagnosed with Clinical hypothyroidism and thyroid-stimulating hormone (TSH) more than or equal to 4.20 mIU/L and between the ages of 20–45 years were enrolled as cases. The control group for the study included female subjects between the ages of 20–45 years and with euthyroid status (TSH levels 0.20–4.20 mU/L). For some tests, the hypothyroidism was further subdivided into subclinical hypothyroidism (TSH: 4.21–10.0 mU/L) and overt hypothyroidism (TSH 10.0 mU/L).

### Exclusion criteria

Any established cases of hypothyroidism or hyperthyroidism on medication or intervention were excluded from the study. Patients with a history of CVD, diabetes mellitus on medication, hypertension, hypercholesterolemia, and pregnancy with euthyroid status and with age below 20 years or above 45 years were excluded from the study.

### Biochemical measurement

The demographic profiles of eighty participants, including age, body mass index (BMI), and blood pressure (mm of Hg), were recorded. The biochemical parameters serum T3, T4, TSH, Total cholesterol (TC), High-density lipoprotein cholesterol (HDL), Triglyceride (TG), and Low-density lipoprotein cholesterol (LDL), hs-CRP, and homocysteine were estimated. The thyroid function was determined by estimating T3, T4, and TSH by the Electro Chemiluminescence method. The normal range for the Thyroid function test was TSH (0.20–4.20 mU/L), T4: 57–148 nmol/L, and T3: 0.9–2.8 nmol/L [13].

The measurement of serum lipids was determined using enzymatic colorimetric tests. Low-density lipoprotein cholesterol (LDL) is calculated by using the Friedewald formula: [LDL=TC-HDL-{TG/5}]. High-Sensitive-C-Reactive Protein was estimated by the Immunoturbidimetric method. Homocysteine was estimated by enzymatic assay based on an enzyme cycling assay principle. Atherogenic indices (AI) are calculated using the below-mentioned formulas: Atherogenic index of plasma (AIP)=log [TG/HDL], Castelli risk index (CI) I=TC÷HDL, Castelli risk index II=LDL÷HDL, Atherogenic coefficient (AEC)={(TC-HDL)÷HDL} [12].

### Data analysis

Data was compiled in an excel file, and SPSS (Statistical Package for Social Science) software version 20.0 was used for statistical analysis. Comparison between groups was analysed using an independent t-test and Mann-Whitney U test based on Parametric and Non-parametric variables. Spearman's rho was used for non-parametric correlations. The summary statistics for the biochemical parameters were reported using mean scores and standard deviations.

## Results

### Demography and study groups

The demographic profile of the 80 participants in the cases and control and their subgroup is depicted in Table 1. The median age of the participants in the control group is 33 years, and 31 years for cases. The median value for systolic blood pressure in both groups was 120 mm of Hg, expressed as median (mm of Hg) and range.

**Table 1:** The comparison of demographic and biochemical profiles among cases and controls *Significant p-value of <0.05<br>NS – Not significant<br>a. Independent samples Mann Whitney U test<br>b. Independent student t-test

Median (IQR)^a^			*p*-value^a^
Parameters	Cases (n=40)	Controls (n=40)	
Age	31 (20–45)	33 (21–45)	NS
BMI (kg/m^2^)	24 (19–41)	24 (16–31)	NS
TSH (mU/L)	8.2 (4.3–68.1)	2.3 (0.8–4.2)	<0.001*
Triglyceride (mmol/L)	1.48 (0.43–4.33)	0.86 (0.50–3.63)	0.001*
HDLc (mmol/L)	1.06 (0.52–2.33)	1.24 (0.70–2.33)	0.015*
hs-CRP (nmol/L)	20 (2–130)	20 (2–115)	NS
Homocysteine (mmol/L)	88.76 (59.1–510.39)	73.23 (37.1–207.12)	0.014*
SBP (mm Hg)	120 (90–160)	120 (100–130)	0.005*
DBP (mm Hg)	80 (60–130)	80 (70–100)	NS
	Mean ± SD^b^		*p*-value^b^
T3 (nmol/L)	0.20±0.022	1.1±0.024	NS
T4 (nmol/L)	86.24±20.21	96.28±18.15	0.02*
Total Cholesterol (mmol/L)	5.62±0.57	4.22±0.67	0.001*
LDLc (mmol/L)	3.65±0.78	2.49±0.65	<0.001*

### Biochemical parameters

TSH, Triglyceride, Total Cholesterol, LDL (Table 1) was significantly elevated in hypothyroid group (cases) when compared to euthyroid group (controls). T4 and HDL were significantly lower in hypothyroid group compared to the euthyroid control group, but the values were within the normal range. Systolic blood pressure (SBP) significantly differed within the hypothyroid group, with the median being within the normal range (median: 120 mm Hg; ranging from 90–160 mm Hg) but did not differ from the control group (median: 120 mm Hg; 100–130 mm Hg). Diastolic blood pressure (DBP) and T3 of both groups were within the normal range with no statistical significance. BMI in both groups had no statistical significance.

Non-parametric independent samples Mann-Whitney U test showed a statistically non-significant mean rank for women with hypothyroidism (42.89) when compared to women with euthyroid status (38.11) (p=0.35), retaining the null hypothesis that the distribution of hs-CRP was the same across both the groups.

Homocysteine showed a statistically significantly higher mean rank for women with hypothyroidism (46.88) than for women with euthyroid (34.13) with a p-value of 0.014, rejecting the null hypothesis that the distribution of homocysteine was the same across both the groups of women.

The participants’ biochemical data were compared between the three subgroups (euthyroid, SCH, and overtHO) (Table 2) by Oneway ANOVA, which was statistically significant for T4, TC, and LDL. In addition, a non-parametric comparison between three groups with the Kruskal-Wallis test showed statistical significance for HDL, TG, and SBP. The comparison between SCH and the control group by parametric independent student t-test showed statistical significance for TC (»p-value« 0.001) and LDL (»p-value« 0.001). However, the non-parametric variables showed a little comparison for age, BMI, and DBP by the Mann-Whitney U test. Table 3

**Table 2:** The comparison of demographic and biochemical profiles among subgroups *significant p-value of <0.05<br>NS – Not significant<br>a. Oneway ANOVA test (between all three groups)<br>b. Non-parametric independent samples Kruskal-Wallis test (between all three groups)<br>c. Independent student t-test (between SCH & Euthyroid)<br>d. Independent samples Mann Whitney U test (between SCH & Euthyroid)

	Median (IQR)^b,d^			*p*-value^b,d^
Parameters	Cases		Controls<br>(n=40)	
	SCH<br> (n=25)	Overt Hypothyroidism<br>(n=15)		
Age	29 (20–45)	36 (22–44)	33 (21–45)	NS^b,d^
BMI (kg/m^2^)	23 (19–35)	25 (23–41)	24 (16–31)	NS^b,d^
TSH (mIU/L)	5.9 (4.2–9.4)	18.5 (10.1–38.8)	2.3 (0.8–4.2)	<0.001^*b,d^
TG (mmol/L)	1.55 (0.58–4.33)	1.53 (0.66–3.93)	0.86 (0.50–3.63)	0.002^*,d^<br>0.005^*,d^
HDLc (mmol/L)	1.09 (0.52–2.33)	1.04 (0.53–1.94)	1.24 (0.70–2.33)	0.04^*,b,d^
hs-CRP (nmol/L)	20 (2–104)	25 (2–130)	20 (2–161)	NS^b,d^
Homocysteine (mmol/L)	88.76 (59.1–185)	88.76 (66.57–510.39)	73.23 (37.1–207.12)	0.031^*,b^<br>0.036^*,d^
SBP (mm Hg)	120 (90–160)	120 (110–140)	120 (100–130)	0.02^*,b^<br>0.01^*,d^
DBP (mm Hg)	80 (60–130)	80 (70–90)	80 (70–90)	NS^b,d^
	Mean ± SD^a,c^			*p*-value^a,c^
T3 (nmol/L)	1.1±0.22	1±0.25	1.1±0.24	NS^a,c^
T4 (nmol/L)	92.67±14.16	75.97±25.10	96.28±18.15	0.04^*,a^<br>NS^c^
TC (mmol/L)	5.46±0.57	5.83±0.52	4.22±0.67	<0.001^*,a,c^
LDLc (mmol/L)	3.52±0.78	3.94±0.67	2.50±0.65	<0.001^*,a,c^

**Table 3:** Details of ROC curve for homocysteine (μmol/mL)

Area	Std. Error	*p*-value	Sensitivity	Specificity	Cut-off point (μmol/L)	
0.669	0.061	0.009	77.5	55	68.05	
Following was a list of cut-offs for the highest sensitivities and specificities						
	Sensitivity			Specificity		
Percentage (%)	97	95	90	97	95	90
Cut-off (μmol/L)	59.18	62.50	65.83	184.19	121.0	108.74

Non-parametric independent samples Mann-Whitney U test for hs-CRP showed an insignificant mean rank comparison between SCH and overtHO groups (»p-value«: 0.586), the comparison between OvertHO (mean rank=31.53) and controls (mean rank=26.68) had no statistical significance (»pvalue«: 0.316). Mean rank comparison among the three subgroups by Kruskal-Wallis showed insignificant p=0.573, indicating that even OvertHO has not produced a change in hs-CRP concentration. Non-parametric independent samples Kruskal-Wallis test for homocysteine concentration distribution among diverse groups of women was statistically significant (»p-value«: 0.031 & 0.036), depicting a significant difference in concentrations dependent on TSH.

### Correlation of study variables

Spearman's rho correlation showed that TSH had a weak positive correlation with hs-CRP (r=0.01, p=0.945) and homocysteine (r=0.146, p=0.363), indicating a statistically insignificant linear relationship. Spearman's rho correlation for controls showed an insignificant, weak negative correlation for both variables.

**Figure 1:**
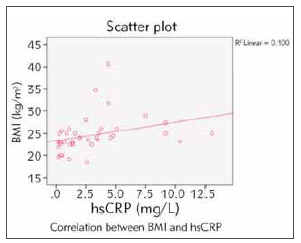
The correlation between BMI and hs-CRP in cases

In cases, Spearman's rho correlation showed a strong significant positive correlation (r=0.544; p=<0.001) between BMI and hs-CRP (Figure 1).

**Figure 2:**
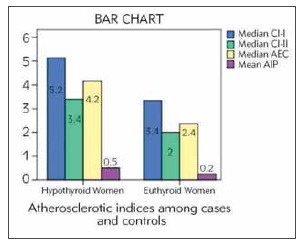
Comparison bar chart for atherogenic indices in women with cases & controls

Median and mean range of various atherogenic indices were significantly higher (p=<0.001) in cases showing the strong influence of hypothyroidism over lipid profile (Figure 2).

### Correlation of atherogenic indices (CI I & II, AIP, and AEC)

Spearman’s rho correlation in cases showed an insignificant positive correlation for AI like atherogenic co-efficient, Castelli risk indices I & II. Controls showed a weak insignificant negative correlation, AEC (r=0.15; p= 0.34); CI-I (r=0.15; p=0.34); CIII (r=0.14; p=0.36). Pearson correlation in cases for AIP showed an insignificant, weak positive correlation (r= 0.02; p=0.86) (Figure 2). Controls showed a weak insignificant negative correlation for above variables.

**Figure 3:**
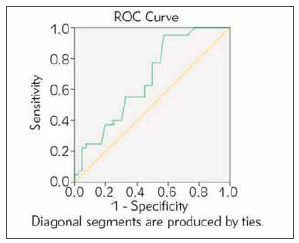
Receiver Operating Characteristic (ROC) curve for homocysteine (μmmol/L)

Percentage frequency and risk stratification of hs-CRP in cases determined that 38% of patients with a high risk (>3) for developing atherosclerosis, the remaining 32% were in intermediate-risk, and 30% were in low-risk categories. In euthyroid female controls, 25% of people were in the high-risk category, 32% in intermediate-risk, and 43% were in the low-risk categories. Serum homocysteine level was utilized to determine CVD risk using the Receiver Operating Characteristic (ROC) curve (Figure 3). The area under the ROC curve was 0.669 (SE:0.061, »p-value: 0.009) (Table 2) (Figure 3).

Framingham Risk Score (FRS) was calculated using an online application. The cases showed a calculated risk of <10% (except for two subjects, the rest of the cases had a <1% risk score), but the risk score in the control group was <1%. Correlation between Framingham risk score and parameters like TSH, hs-CRP, homocysteine, lipid profile, and atherogenic indices in cases and controls were statistically insignificant, except for TC, where the cases showed a significant correlation (TC: r=0.342, p=0.031 and r=0.389, p=0.013).

## Discussion

In this cross-sectional case-control study, we tried to investigate the influence of thyroid hormone concentration in premenopausal women on the blood levels of homocysteine, hs-CRP, and atherogenic indices, which can determine the risk of developing cardiovascular diseases. Our findings indicate that thyroid hormone dysfunction can influence the concentrations of biochemical parameters used to assess the risk of developing cardiovascular diseases.

Findings in our study indicate that the median age of participants in the overt hypothyroid group had a higher median age (36 years) compared to the age-matched cases, implying that hypothyroidism severity increases with age. Similar findings were reported by Surks et al. [14] the prevalence of SCH in an age-specific manner with the NHANNES III survey indicated that the TSH range increased with age, 1.8% participants in 30–39 years, 2.4% participants in 40–49 years, and 3.5% participants in 50–59 years of age showed TSH 4.5 μIU/mL.

The BMI in this study did not show much variation between the controls (24 kg/m^2^), SCH (23 kg/m^2^), and OvertHO (25 kg/m^2^). The overt hypothyroidism group had a higher BMI. This can be due to the possible effect of dysfunction in the hypothalamic-pituitary-adipocyte axis and its effect on the TSH and pituitary hormone receptors on adipocytes. Comparable results are reported by Milionis et al. [15]. They reported a significant relationship between BMI and thyroid status (*P* value 0.037 and < 0.001 for TT3 and TT4), and Rajini et al. [16] reported a strong correlation between TSH levels and BMI (TSH levels 26.30±21.4 mU/L and 61.66±27.50 mU/L in overweight and obesity categories). The significant finding in this study was the presence of dyslipidaemia in most cases compared to the controls, with a significant reduction in HDL concentration. Dyslipidaemia in thyroid dysfunction has been previously established by Hussain et al. [17] (TC: 5.57±1 (±38.75) mmol/L in SCH versus 4.52±0.75 mmol/L in normal patients), and Hueston et al. [18] reported significant dyslipidaemia in hypothyroidism (5.85 mmol/L (44.2) versus 5.62 mmol/L (43.7)). The thyroid hormone regulates lipogenesis and lipolysis through its receptors (α and β) on the liver and white adipose tissue. These functions are deranged during thyroid dysfunction [15] [17] [16].

Hs-CRP is an inflammatory marker, this marker did not show significant variation in the three groups, but it was higher in the overt HO group, which depicts the increase in the inflammatory process concurrent with the progression of the disease. Aksoy et al. [19] observed similar results with no significant difference in hs-CRP concentration between subclinical and euthyroid groups (1.20 mg/L (0.73) versus 1.63 mg/L (0.344), p=0.125). Other authors like Tuzcu et al. [20] reported a median hs-CRP concentration significantly higher in hypothyroidism patients compared to the euthyroid population (4.2 +/- 0.8 mg/L SCH and 1.05+/-0.3 mg/L, respectively, p=0.0001).

Earlier studies have linked the increased hs-CRP in hypothyroidism with the emergence of dyslipidemia and act as an inflammatory marker that plays a role in forming atherosclerotic plaque by encouraging monocyte recruitment and increased nitric oxide release, which leads to endothelial dysfunction. Our study did not show any significant association between the concentration of hs-CRP and serum lipids except for the serum TG. Hyperhomocysteinemia is considered when the homocysteine concentration in the blood is above 15 μmol/L. Our study demonstrated a significant difference in homocysteine concentration between cases and control. Similar findings have been observed by Dong et al. [21] in their research (96.20±55.0 versus 62.9±19.20 μmol/L, *p* <.01). Homocysteine is known to promote atherogenesis by increasing oxidative stress and promoting the formation of oxidized LDL. The other exciting finding observed in this study was a significant correlation between BMI and hs-CRP in hypothyroid patients. This indicates the presence of the obesity-related latent inflammatory process in the patients with high BMI and further promotes the production of inflammatory cytokines like TNF-α, interleukins-6 (IL-6), and IL-1 in adipose tissue. Other authors like Tamakoshi et al. [22] have also established similar findings in their study (OR for CRP is 1.83 (95% CI: 1.25–2.69), 2.63 (1.69–4.11), and 10.31 (2.17–48.98) for normal, overweight, and obese categories [21].

The other significant finding established in the study was the higher atherogenic indices observed in cases compared to controls. Specifically, the AIP high-risk category was significantly higher in cases when compared to controls. A similar result has been observed by Bhardwaj et al. [23] (AIP: 0.39±0.03 versus 0.09±0.02, p<0.001). We observed that the overt hypothyroid group presented significant dyslipidemia, and higher hs-CRP but with an average homocysteine concentration. Similar findings have been observed in a study conducted by Singh et al. [24] (TC, LDL-C, TG (P<0.01, P<0.001, and P<0.05) in SCH). The ROC curve for homocysteine established a cut-off range of 68.5 μmmol/L cut-off, which is within the normal reference range. The area under the curve is 0.669, which is an insignificant finding and indicates that homocysteine can be a fair to poor test. Shih et al. [25] reported similar results for the area under the ROC curve (Curve area ROC: 0.67). This analysis suggests that homocysteine estimation alone may not be sufficient to differentiate female patients with atherogenic risk from those without the atherogenic risk in hypothyroid and euthyroid states. The FRS calculation in our study indicated that the risk of developing CVD in the next ten years for the study participants was low. Similar findings have been observed by Rodondi et al. [26] who reported that in the growing elderly population, FRS underemphasized the CVD risk, which was observed in around 51% of female study participants and failed in risk prediction by using the other variables with FRS. Sara et al. [27] reported that the FRS poorly distinguished the secondary event (long-term) risks like infarction (C-statistic: cardiac death and MI, 56.8), death due to a cardiac cause in the patients who have undergone percutaneous coronary intervention procedure (*P*=51.3) [24] [27] [17].

## Conclusions

The primary findings observed in this study are higher BMI in the overt hypothyroidism, dyslipidaemia, and higher concentration of homocysteine and hs-CRP. The positive association between BMI and serum hs-CRP concentration indicates the inflammatory process initiated in the tissues due to the chronic effects of obesity. This is assisted by the enhanced production of inflammatory markers like cytokines TNF-α, IL-1, and IL-6. Also, we have established a role played by advancing age on the status of thyroid dysfunction, the implication being that the severity of hypothyroidism increases with age. Dyslipidaemia observed in cases with hypothyroidism is due to the metabolic derangements in functions of insulin and increased oxidative stress in the body. Patients with hypothyroidism are at increased risk of developing atherosclerosis. This is related to the increase in the concentration of hs-CRP and homocysteine in the blood and tissues, which are known to initiate the inflammatory process by encouraging monocyte recruitment, increased expression of adhesion molecules like plasminogen activator inhibitor-1, increased production of oxidized LDL and change in the macrophage engulfing process of oxidized LDL. Our study adds further evidence to the debate on an association between thyroid dysfunction and atherosclerosis. The atherogenic indices also establish a higher risk of CVD in cases compared to controls. This study provides compelling evidence that premenopausal women with hypothyroidism are more predisposed to develop cardiovascular disease than their euthyroid counterparts. All newly diagnosed female hypothyroid patients need to be screened for markers like homocysteine and atherogenic indices to predict the risk of cardiovascular disease.

## Dodatak

### Limitations

The major drawback of the study was the lack of financial resources to test a larger sample size. Age-matched healthy men could have been included in this study as controls to strengthen the study findings, and the utilization of fT4 could have enhanced the difference between SCH and overt hypothyroidism were the study's limitations. The study can be expanded to a larger population of men and women, including postmenopausal women in different age groups, to predict atherosclerosis risk early. Furthermore, a prospective study with male controls and cases may be conducted to determine the impact of hypothyroidism treatment on CVD risk.

### Acknowledgments

The authors express sincere gratitude to the Dean, Kasturba Medical College, Manipal, Manipal Academy of Higher Education, for providing us with suitable facilities and continuous encouragement to do research. We acknowledged the inputs for reviewing results and discussion from Dr. Krishnananda Prabhu R V, Professor, and other faculty from the Department of Biochemistry Kasturba Medical College, Manipal.

### Funding

This work was supported by Intramural grant funding from Kasturba Medical College, Manipal Academy of Higher Education, India.

### Contribution

The authors' RM, VB, VSB, VJ, and BMG conceptualized the study. VB, RM, and VJ wrote the first draft. RM, VB, VJ, and VSB analyzed and interpreted the data. The authors (RM, VJ, BMG, VB, and VSB) contributed to its administration, discussion, conclusion, and critical revision. All authors approved the final manuscript for submission.

### Data availability

The data generated during the research and analysis are not publicly available but from the corresponding author on a reasonable request.

### Conflict of interest statement

All the authors declare that they have no conflict of interest in this work.
